# Multiple Wedge in Dilating Urethral Stricture

**Published:** 1875-04

**Authors:** John S. Coleman

**Affiliations:** Augusta, Ga.


Gradual Dilatation of Stricture of the Urethra on the Principle of the "Multiple Wedge."—*By John S. Coleman, M.D., Augusta, Ga.*—As both internal urethrotomy and divulsion are occasionally followed by such serious consequences, a new method of general dilatation must meet the approval of those members of the profession who treat cases of stricture. A gentleman applied to me for relief from retention of urine. Failing to introduce an ordinary catheter, the stricture being in the membranous portion of the urethra, I took one of Gouley's whalebone guides and passed it into the stricture, but not into the bladder. The effort relaxed the spasm sufficiently to admit of his voiding his urine. With the view of eliminating this element of trouble, I directed him to make use of an enema of thirty drops of laudanum just before his next visit. (It is my habit to use quinine freely during the treatment of stricture, as a prophylactic against urethral fever.) On the third day he returned, and I readily introduced into his bladder one of the whalebone guides; after allowing it to remain a few moments the idea occurred to me that I might pass another down by its side, which, with some difficulty, I suceeeded in accomplishing. At the third sitting I introduced four of the guides. At the fourth I introduced two guides, together with three gum-elastic bougies of Gemrig's scale, Nos. 3, 4, and 6, the whole equalling in bulk about a No. 7, English scale. On a previous occasion, in a case of retention, I succeeded in relieving the patient by the simple introduction of one guide.

In reply to my communication to Dr. Gouley on the subject, he says: "I have myself wedged in two and sometimes three of these bougies side by side, and have thus rendered otherwise impassable strictures amenable to ordinary dilatation. Three years ago I had some whalebone probe-pointed bougies made very small (capillary) for the first two inches from the vesical end, and thence increasing gradually to Nos. ½, 1, 2, 3, and 4, so that I could accomplish more dilatation with a simple bougie at one sitting than I could with two or three ordinary whalebone capillary bougies introduced side by side."

In closing his letter he says: "I would suggest also that a small tunnelled sound or catheter be used after having passed three or four capillary instruments, and while they are still in position. In this manner the wedge-principle could be carried out to the point of divulsion if desirable."

It is quite evident to my mind that Dr. Gouley, though he had used the "two or three guides side by side," did not appreciate the principle involved, for he abandoned it for the "whalebone probe-pointed bougies." The name of this little whalebone instrument designates the use for which it is intended by Dr. Gouley, viz: a "guide" upon which to pass a "tunnelled grooved sound" into a stricture, to facilitate the operation of external urethrotomy. My plea is that the "wedge-principle" will obviate, in most cases, the necessity for divulsion, internal or external urethrotomy. I therefore claim priority, if not in actual use, in first appreciating and making known the multiple-wedge principle in the treatment of urethral stricture. I am aware that many medical men will at once say that there is nothing new in the wedge; that all conical bougies are wedges. This I grant, but claim that it is a new application of the *principle* of the wedge in the treatment of stricture.

It ought to be a self-evident proposition that it is easier to introduce singly the component parts of a wedge than to introduce it as a whole. Take, for example, a No. 12 conical bougie, even capillary at its point, and attempt to introduce it into a stricture, where is the point of friction and resistance? Is it not around the entire circumference of the stricture?

Diminish the size of the instrument and in a direct ratio you diminish the amount of resistance. After having passed into a stricture one of these whalebone guides, the second has to overcome the friction and resistance of but one-half the circumference of the stricture and the *line of contact* with the other bougie.

Now that we have passed these two, we have a groove in front and behind them, through which we can readily pass the third and fourth, having now to overcome the resistance of but one-fourth the circumference of the stricture and the *two lines of contact* with the instruments already in position.

For these guides the profession owe Dr. Gouley a lasting debt of gratitude.—*Medical Record.*

